# Shaping skills of mental hygiene and psychologically verified behavioral techniques under the situation of the pandemic

**DOI:** 10.1192/j.eurpsy.2021.820

**Published:** 2021-08-13

**Authors:** N. Burlakova, V. Oleshkevich

**Affiliations:** 1 Department Of Neuro- And Pathopsychology, Lomonosov Moscow State University, Moscow, Russian Federation; 2 Academic Department, Scientific-Practical Children’s Mental Health Centre n. a. G. Sukhareva of Moscow City Department of Healthcare, Moscow, Russian Federation

**Keywords:** Mental Hygiene, COVID-19, Behavioral Techniques, Psychotechniques

## Abstract

**Introduction:**

COVID-related situation has produced multiple challenges in the field of implementation of the restrictions.

**Objectives:**

Russian media, NGOs and sociological institutions collected and processed data on following the COVID-related guidelines in Moscow and in other regions. According to these data, different social groups behave differently in respect to the restrictions.

**Methods:**

The following methods were used: analysis of information in media, interviews with child psychologists and pedagogues, personal involved and non-involved observation.

**Results:**

The COVID-related restrictions are often violated in all the Russian provinces. Observation and interviews demonstrate similar results. Young people aged 13–20 transgress the regulations most often. The reasons for that are not only the insufficient information, but also neurotic reactions and respective behavior: suppression, reaction formations, reactions of denial. Moreover, new behavioral norms (e.g. wearing masks) are insufficiently shaped yet, which makes following the new rules even more difficult. Furthermore, during the introduction of those regulations, their possible pathopsychological consequences were not taken into consideration, e.g. consequences of isolation, maintaining social distance, communication while wearing masks, fears, stress, paranoic reactions.

**Conclusions:**

The pathopsychological consequences of introduced measures should be taken into consideration. Moreover, the phrasing of regulations needs reshaping and implementation of techniques of mental hygiene to prevent the development of mental disorders. Efficient shaping of respective skills might help to increase the percent of people following the guidelines too. However, all those issues require additional research.

